# Enhanced Anaerobic Digestion of Long Chain Fatty Acid by Adding Magnetite and Carbon Nanotubes

**DOI:** 10.3390/microorganisms8030333

**Published:** 2020-02-27

**Authors:** Alsayed Mostafa, Seongwon Im, Young-Chae Song, Seoktae Kang, Dong-Hoon Kim

**Affiliations:** 1 Department of Civil Engineering, Inha University, 100 Inha-ro, Nam-gu, Incheon 402-751, Korea; ama_mostafa@ymail.com (A.M.); deback3838@naver.com (S.I.); 2 Department of Environmental Engineering, Korea Maritime and Ocean University, Busan 49112, Korea; soyc@kmou.ac.kr; 3 Department of Civil and Environmental Engineering, KAIST 291 Daehak-ro, Yuseong-gu, Daejeon 34141, Korea; stkang@kaist.ac.kr

**Keywords:** long-chain fatty acid, direct interspecies electron transfer, anaerobic digestion, magnetite, carbon nanotubes

## Abstract

This study investigated the impact of stimulating direct interspecies electron transfer (DIET), by supplementing nano-sized magnetite (nFe_3_O_4_, 0.5 g Fe/g VSS) and carbon nanotubes (CNT, 1 g/L), in anaerobic digestion of oleic acid (OA) at various concentrations (0.10–4.00 g chemical oxygen demand(COD)/L). Both supplementations could enhance CH_4_ production, and its beneficial impact increased with increased OA concentration. The biggest improvements of 114% and 165% compared to the control were achieved by nFe_3_O_4_ and CNT, respectively, at OA of 4 g COD/L. The enhancement can be attributed to the increased sludge conductivity: 7.1 ± 0.5 (control), 12.5 ± 0.8 (nFe_3_O_4_-added), and 15.7 ± 1.1 µS/cm (CNT-supplemented). Dissolved iron concentration, released from nFe_3_O_4_, seemed to have a negligible role in improving CH_4_ production. The excretion of electron shuttles, i.e., humic-like substances and protein-like substances, were found to be stimulated by supplementing nFe_3_O_4_ and CNT. Microbial diversity was found to be simplified under DIET-stimulating conditions, whereby five genera accounted for 88% of the total sequences in the control, while more than 82% were represented by only two genera (*Methanotrix concilli* and *Methanosarcina flavescens*) by supplementing nFe_3_O_4_ and CNT. In addition, the abudance of electro-active bacteria such as *Syntrophomonas zehnderi* was significantly increased from 17% to around 45%.

## 1. Introduction

Besides having various advantages such as being 10^6^ times faster than traditional interspecies hydrogen transfer, direct interspecies electron transfer (DIET) features potential for both conserving the cell energy and securing a condensed electron flow [1,2,3]. Specifically, providing a DIET-based environment avoids the energy consumption dedicated for the generation of extracellular conductive pili and associated c-type cytochromes, which are required for the cells’ electrical connection during anaerobic digestion (AD) [4,5]. Through DIET stimulation, CH_4_ production rates and yields when treating various wastes have been enhanced [4,5,6,7]. However, research on the capability of DIET-stimulated AD systems to handle toxic substrates is still limited.

Efficient AD of long chain fatty acids (LCFAs) is needed for the valorization of complex wastes such as lipid-containing wastes, which has higher CH_4_ potential than that produced from other substrates such as proteins and carbohydrates [8,9,10]. However, LCFAs can inhibit and/or destabilize the AD process by adsorbing onto the surface of methanogenic consortia, inhibiting nutrient transfer [11,12]. To alleviate the inhibition, the addition of chemicals such as CaCl_2_ and NaOH has been attempted to increase LCFAs’ solubilization [13,14,15,16]. However, the achieved improvements were limited, showing less than 10% increase compared to the control [15]. The main hurdle of anaerobic LCFA biodegradation is known as syntrophic collaboration between acetogens and methanogens [17,18]. This syntrophic collaboration can be significantly improved via stimulating DIET reaction, which can secure a more favorable thermodynamic route, than conventional route, for LCFAs degradations [19,20].

Previously, it was stated that the supplementation of conductive materials (CMs) in AD systems can play a vital role in stimulating DIET [21,22]. The utilized CM can be classified into carbon-based CM (CBCM) and iron-based CM (IBCM). Importantly, in the presence of CMs, DIET could be stimulated without the need for electrically conductive pili (e-pili) and outer-surface c-type cytochromes [2]. Various CM, e.g., magnetite (Fe_3_O_4_) [23], carbon cloth [24], granular activated carbon (GAC) [25], biochar [26], ferro/ferric oxide [27], and carbon fiber [28] were supplemented to AD systems and brought about improved CH_4_ production rates and yields. Based on their electrical conductivity properties and ability for long-range electron exchange, CMs could work as electron conduits between syntrophic and methanogenic consortia [29,30].

Since IBCM contains mainly iron, there is a probability for the release of iron ions from IBCM during the AD process. However, it is still unclear whether the concentration of the released iron is effective and implicated in the enhancing impact played by supplemented IBCM or not. Two possibilities were discussed in the literature: firstly, the concentration of the released iron ions was significant and enriched the organics’ degraders, being responsible for the enhanced substrate degradation efficiency [31,32]. Secondly, the concentration of the released iron ions is negligible, and the enhancing effect, acquired by IBCM, has to be principally ascribed to the electrical conductivity of IBCM [33]. Therefore, more research effort must be implemented in order to clarify the mechanism behind the IBCM-caused performance enhancement and comparing it to that the mechanism in CBCM.

The aim of this study was to investigate the impact of DIET stimulation on AD treating oleic acid (OA), which was utilized as a model LCFA. OA is the most abundant LCFA in wastewater [34]. Targeting such DIET stimulation, batch tests were performed in the presence of nano-sized magnetite (nFe_3_O_4_, 0.5 g Fe/g VSS) or carbon nanotubes (CNTs, 1.0 g/L) and compared with control (without CM supplementation). The tested range of OA concentrations was 0.10–4.00 g chemical oxygen demand (COD)/L. Based on iron concentration and sludge conductivity observations, a mechanism of action for both of nFe_3_O_4_ or CNT was hypothesized. In addition, the microbial community was analyzed by using a next generation sequencing (NGS) tool. This research is expected to provide a new insight into the AD of toxic substrates via DIET stimulation.

## 2. Materials and Methods

### 2.1. Inoculum Preparation

Mixed culture of anaerobic digester sludge, utilized as a seed inoculum source herein, was sampled from the local sewage treatment plant. After sampling, the mixed culture was filtered using a sieve (pore size of 2 mm), to remove big suspended solids. The pH, total suspended solids (TSS), volatile suspended solids (VSS), and chemical oxygen demand (COD) of inoculum were 7.5, 18.1 g/L, 15.0 g/L, and 21.6 g/L, respectively. For releasing any residual biogas in the sludge, it was precultured in an incubator at 37 ± 0.1 °C for three weeks.

### 2.2. Experimental

Batch tests were carried out in serum bottles having 270 mL of total volume (effective volume = 200 mL). For each serum bottle, a volume of 107 mL of inoculum was added in order to ensure a VSS concentration of 8 g VSS/L. Pure OA (99.0%), utilized as a sole carbon source, was added to the batch bottles to reach the concentrations of 0.10, 0.25, 0.50, 1.00, 2.00 and 4.00 g COD/L. For investigating the impact of DIET stimulation upon OA methanation, certain concentrations of nFe_3_O_4_ and CNT were added into corresponding batches. In specific, for each of nFe_3_O_4_-supplemented batches, 1.1 g of nFe_3_O_4_ was added, in order to reach a concentration of 0.5 g Fe/g VSS [20]. The utilized nFe_3_O_4_, purchased from Sigma–Aldrich (St. Louis, MO, USA) had a particle size of 50-100 nm. To each of the CNT-supplemented batches, 0.2 g of CNT was added in order to reach a concentration of 1 g CNT/L [1]. Batches with neither nFe_3_O_4_ nor CNT were operated and considered as control. Nutrients of NH_4_Cl, KH_2_PO_4_, and FeCl_2_·4H_2_O were added for all batches in order to provide COD:N:P:Fe ratio of 100:5:1:0.33. Further, the following nutrients (in mg/L): NaHCO_3_ (1000); MgCl_2_·6H_2_O (100); CaCl_2_·2H_2_O (75); Na_2_MoO_4_·4H_2_O (0.01); H_3_BO_3_ (0.05); MnCl_2_·4H_2_O (0.5); ZnCl_2_ (0.05); CuCl_2_ (0.03); NiCl_2_·6H_2_O (0.05); CoCl_2_·2H_2_O (0.5); Na_2_SeO_3_ (0.05) were also added [35]. Initial pH values for all bottles were set at 7.5 ± 0.1, using 3 M HCl or 3 M NaOH. All bottles were flushed using 99.99% N_2_ gas for 10 min in order to provide anaerobic condition. Afterwards, bottles were sealed using butyl rubber stoppers, and were placed in an incubator at agitation speed of 150 rpm and temperature of 37 ± 0.1 °C. Biogas volume and composition were analyzed periodically until the amount of biogas production was negligible. All experiments were implemented in duplicate, and the results were averaged.

### 2.3. Analysis and Calculations

COD, TSS, VSS and pH were measured according to Standard Methods [36]. For getting the contents of methane (CH_4_) and CO_2_, sampling was done from the headspaces of the batches using a gas-tight micro syringe. Then, samples were analyzed using a gas chromatograph (Gow-Mac Series 580, Bethlehem, PA, USA) equipped with a thermal conductivity detector and a 1.8 m × 3.2 mm stainless-steel column. The temperatures of injector, column, and detector were kept at 50, 80, and 90 °C, respectively. The carrier gas was N_2_ and the flow rate was 30 mL/min. For excitation-emission matrix (EEM) analysis. Forty mL of the supernatant, harvested from 2 g COD/L batches, were filtered through 0.45 µm filter papers. After normalization of COD concentration to 60 mg/L, EEM profiles were examined using fluorescent spectroscopy (Shimadzu RF530, Tokyo, Japan) at excitation wavelengths from 220 nm to 380 nm and emission from 250 nm to 600 nm, as previously suggested [37]. For conductivity measurement, sludge samples were collected at the end of experiment from the batches of 2 g COD/L and centrifuged for 5 min at 8000 rpm, then washed using 0.1 M NaCl solution. Afterwards, sludge conductivity was measured based on three-probe electrical conductance measurement [1]. The concentration of iron ions in the mixed liquor of 2 g COD/L batches was analyzed using atomic absorption spectrometer (AAnalyst 400, PerkinElmer, Spokane, WA, USA). The reason to take samples of sludge conductivity, iron ions and EEM from 2 g COD/L batches was that such concentration witnessed the best performance, in terms CH_4_ yield, among all batches. The experiment was carried out for two times, and the average values were given.

A modified Gompertz model (Equation (1)) has been applied for calculating the lag period, CH_4_ production rate and potential.

(1) $$M_{\left(t\right)}=M_{0}\;\times \;\exp\left\{-\exp\left[\frac{R_{0}e}{M_{0}}\left(\lambda -t\right)+1\right]\right\}$$

where M_(t)_ represents the cumulative CH_4_ production at incubation time t (mL); *M*_0_ is the CH_4_ production potential (mL); *R*_0_ refers to the CH_4_ production rate (mL/d) and λ is the lag period (d), considering that the e value is 2.71828.

### 2.4. Microbial Community Analysis

The mixed liquors of 2 g COD/L batches were analyzed by using a NGS tool for determining the populations of bacteria and archaea. Using Soil DNA Kit and Ultraclean Microbial DNA Isolation Kit (Mo Bio 21 Laboratories, Carlsbad, CA, USA), DNA of the samples was extracted and purified. Then, the emPCR amplification steps were carried out, as previously described [38]. The 16S universal primers 27F (5′GAGTTTGATCMTGGCTCAG 3′) and 800R (5′ TACCAGGGTATCTAATCC 3′) for bacteria; Arch349-F (5′ GYGCASCAGKCGMGA AW 3′) and Arch1017R (5′ GGC CAT GCA CCW CCT CTC 3′), were utilized for the amplification of the 16s rRNA genes obtained [39]. The PCR reaction mix was then purified using AMPure beads (Beckman Coulter, Danvers, MA, USA). Thereafter, sequencing was performed using a 454 pyrosequencing Genome Sequencer FLX Titanium system (Life Sciences, Branford, CT, USA), according to the manufacturer’s instructions, at a commercial sequencing facility (Macrogen, Seoul, Korea). Using the software MOTHUR for analyzing the sequences generated from pyrosequencing, identification of the operational taxonomic units (OTUs), taxonomic assignment, community comparison, and statistical analysis could be done. For avoiding the consequences of poor sequence quality and sequencing potential errors, filtration and trimming of sequence were implemented, as previously described [38]. The sequences spanning the same region were then realigned using the NCBI BLAST database (www.ncbi.nlm.nih.gov). In the database screening with the BLAST program, the threshold E-value to include a sequence in the next iteration was 0.001.

## 3. Results and Discussion

### 3.1. CH_4_ Production At Different Concentrations of Oleic Acid

**Figure 1:**
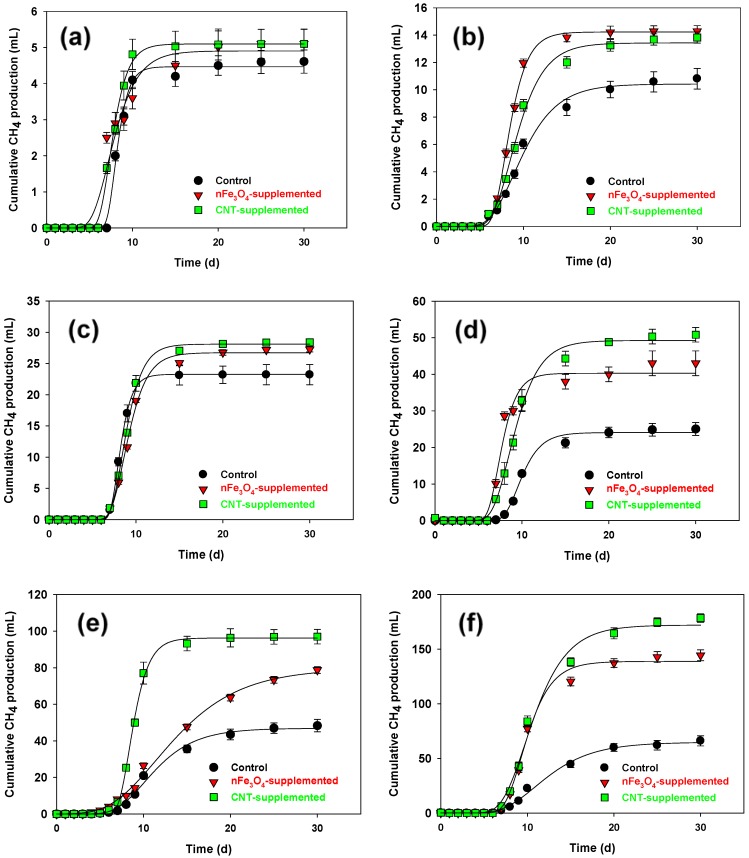
Effect of supplementing nFe_3_O_4_ and CNT on cumulative CH_4_ production from oleic acid at concentrations (g COD/L) of (**a**) 0.10, (**b**) 0.25, (**c**) 0.50, (**d**) 1.00, (**e**) 2.00, and (**f**) 4.00.

Figure 1 presents the impact of supplementing nFe_3_O_4_ and CNT on cumulative CH_4_ production from various concentrations of OA. CH_4_ production curves were fitted by using the modified Gompertz equation (R^2^ > 0.99) (Table 1). No significant difference was found in the lag period among all batches. It was easy to observe that supplementation with both nFe_3_O_4_ or CNT enhanced CH_4_ production from OA, whose beneficial impact increased with increased OA concentration, and the biggest improvements of 114% and 165% compared to the control were achieved by nFe_3_O_4_ and CNT, respectively, at OA of 4 g COD/L. This was due to the well-known toxic effect of OA, and previous studies reported that at OA concentration of 3.55 mM (2.91 g/L), 50% of the relative methanogenic activity was lost [40,41,42]. On the other hand, DIET stimulation, acquired by CM supplementation, might secure a more thermodynamic routes for the degradations of OA than that conventionally followed indirect electron transfer via hydrogen (Equations (2) and (3)).

C_18_H_33_O_2_^−^ + 16H_2_O → 9CH_3_COO^−^ + 15H_2_ + 8H^+^ ∆*G* = +340.9 kJ/mol (2)

C_18_H_33_O_2_^−^ + 16H_2_O → 9CH_3_COO^−^ + 38H^+^ + 30e^−^ ∆*G* = −641.1 kJ/mol (3)

It also seemed that the achieved enhancement in digestion performance by CNT was higher than that obtained by using nFe_3_O_4_, at all tested OA concentrations, with sole exception of 0.25 g COD/L. The highest CH_4_ yield was obtained at 2 g COD/L, whereas yield values (calculated based on the fact 1 g COD is equal to 350 mL CH_4_) were 33.5 ± 2.3, 56.9 ± 3.5 and 68.7 ± 5.4% for the control, nFe_3_O_4_-supplemented, and CNT-supplemented batches, respectively. The reason for the CNT superiority could be related to the conductivity of the formed microbial aggregate and the type of electron shuttles utilized, which can work along with DIET pathway. This will be discussed in detail in Section 3.2. Our CH_4_ yield increments were much higher than those noticed in glycerol-treating batches, where the maximal yield improvement, achieved by Fe_3_O_4_ supplementation, was 22.2% [20]. Further, a CH_4_ yield of 29.5% could be obtained by adding rusty scrap iron containing different types of ferric oxides, when treating waste activated sludge [43]. Additionally, CNT could boost the butyrate conversion to CH_4_ by 1.5 times [44]

At relatively low concentrations of OA (0.10, 0.25 and 0.50 g COD/L), the enhancements achieved by supplementing nFe_3_O_4_ or CNT are not high, probably, because these concentrations are tolerable for microbial consortia [40,41,42]. However, when the OA concentration exceeded this threshold, the positive impact of added CM starts to be obvious. The range of LCFA concentrations that cause inhibition for the microbial community, was found to be 1–5 g/L; other studies, however, mentioned that lower concentrations of LCFA can also be toxic [8,45]. Therefore, it can be stated that the DIET stimulation merit can be more apparent under harsh conditions. CH_4_ production rate, observed in nFe_3_O_4_, fluctuated with OA concentration, showing two peaks of 12.5 and 26.8 mL/d, observed at OA concentrations of 1.00 and 4.00 g COD/L. On the other hand, the CH_4_ production rate achieved by control and CNT-supplemented batches peaked at 9.8 and 28.6 mL/d, when the OA concentration was 0.50 and 2.00 g COD/L, respectively. Comparatively, a previous study found the concentration of 1.9 g COD/L, showed a CH_4_ production rate of 11.04 mL/d, while using the treatment of OA and acetate using anaerobic granular sludge that was bioaugmented with *Syntrophomonas zehnderi*.

Although the DIET pathway stimulation impact achieved by nFe_3_O_4_ and CNT is mainly depending on their conductivity features, they showed different CH_4_ production improvements, therefore, we report the impact of these CM on both the broth characteristics and microbial community structure in the following section.

### 3.2. Conductive Material-Based Broth Change

In order to observe the changes in the AD broth caused by the supplemented nFe_3_O_4_ and CNT, three parameters were taken into consideration, i.e., sludge conductivity, iron concentration, and the distribution of soluble microbial metabolite (SMP) that was revealed through the EEM spectrum. The sludge conductivity was found to be increased from 7.1 ± 0.5 (control) to 12.5 ± 0.8 (nFe_3_O_4_-added) and 15.7 ± 1.1 µS/cm (CNT-supplemented), which can be ascribed to the high conductivity properties of these materials [46,47]. Such properties allow DIET establishment among syntrophic partners and methanogens [48]. Enhanced sludge conductivity was proven to have a direct relation with electron transport efficiency, in AD broth, which reflects on CH_4_ production [49]. Electrical aggregates are recently denoted as one of DIET occurrences [50]. The sludge conductivity values here are comparable to those mentioned in a previous study that denoted enhancements in sludge conductivity by 91% and 37% as a result of supplementing nitrogen-doped sewage sludge carbon and granular activated carbon, respectively [51]. Also, the supplementation of Fe_3_O_4_, where the final iron concentration was 1.1 g/L, into an up-flow anaerobic sludge blanket (UASB) reactor treating sulfate-rich wastewater raised the sludge conductivity by 194% [52]. Although, the CNT-supplemented batches contained only 0.2 g of CNT, they could cause higher sludge conductivity that that achieved by 1.1 g nFe_3_O_4_ found in nFe_3_O_4_-supplemented batches. CNT is, indeed, more effective than all other CBCMs, in regards to DIET stimulation [1,53]. On the other hand, CNT had almost similar electric conductivity as nFe_3_O_4_ (>10,000 S/m) [46,54], therefore the higher sludge conductivity after CNT supplementation, compared to nFe_3_O_4_, might be because of the difference in specific surface area that reached 948 and 101 m^2^/g in CNT [55] and nFe_3_O_4_ [56], respectively. Further, the superiority of CNT over nFe_3_O_4_, in terms of enhancing the sludge conductivity, might be correlated with the results shown in Figure 1. Additionally, CNTs might be implicated in efficient anaerobic cell attachment and proliferation, as well as enhanced microbial growth [57].

The concentration of iron ions (Fe^3+^ and Fe^2+^), found in the supernatant of batches under OA concentration of 2.0 g COD/L, was measured. Results showed that the iron ion concentrations were 10.1 ± 0.9, 42.5 ± 3.9, 14.2 ± 1.3 mg/L, for control, nFe_3_O_4_- and CNT-supplemented batches, respectively. The detection of iron, in the ionic form, in the control batches might be because of small amounts of iron in the seed sludge, while the reason in the case of CNT-supplemented batches might be the presence of iron impurities in the CNT. In both cases, this small value of iron concentration is not expected to affect the AD performance. On the other hand, higher iron concentrations, found in nFe_3_O_4_-batches, might have an impact on the digestion process. Specifically, the release of iron ions was found to enhance the activity of iron-reducing bacteria (IRBs), resulting in increased CH_4_ yield [31,32]. Therefore, it might not be accurate to attribute the enhanced CH_4_ yield (Figure 1) solely to the electric conductivity of nFe_3_O_4_. Previously, when ferric oxyhydroxide was added to a continuously stirred tank reactor treating waste activated sludge, increases in iron ions concentration by 1.3–2 fold were observed [32]. Although, the iron concentration found in the nFe_3_O_4_-supplemented batch was 2.8 times higher than that in CNT- supplemented batch, this was not reflected in the CH_4_ generation profile. The reasons could be that a portion of the released iron ions might react with OA, forming iron-oleate [58], or the increment of iron concentration from 10 mg/L to 50 mg/L was not enough to have an impact on the performance enhancement [59,60].

**Figure 2:**
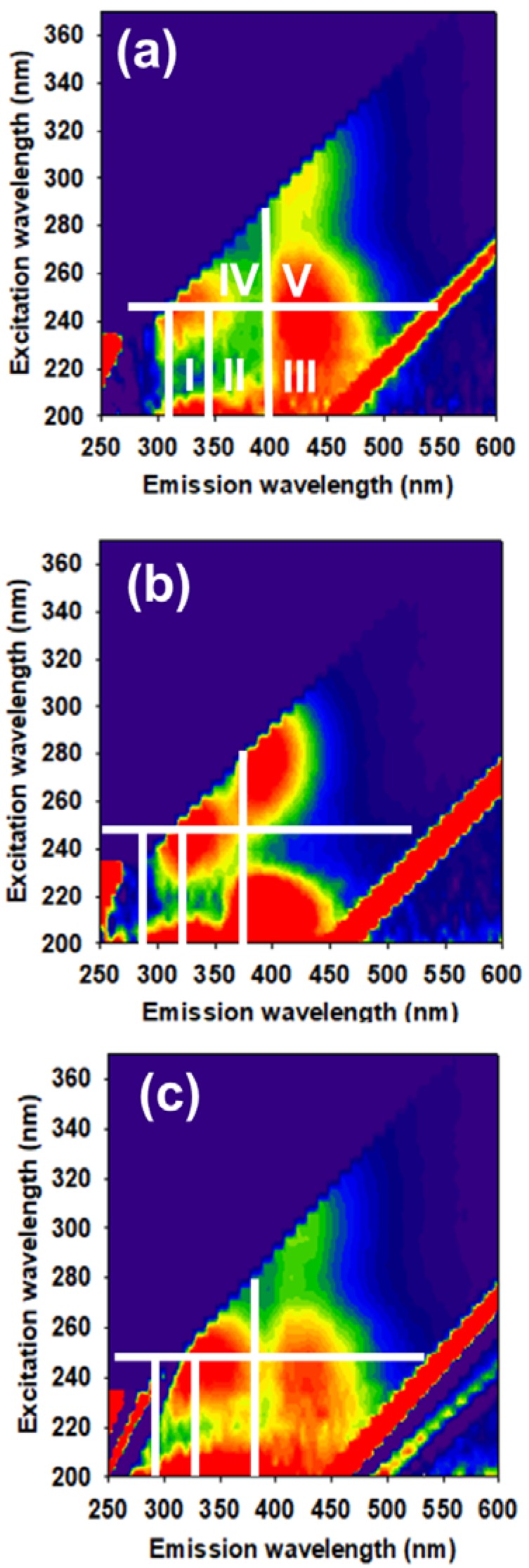
EEM spectra for (**a**) control, (**b**) nFe_3_O_4_-supplemented, and (**c**) CNT-supplemented digestion broth at concentration of 2.0 g COD/L.

Figure 2 shows the qualitative EEM spectra of the supernatant, harvested from control, nFe_3_O_4_-supplemented and CNT-supplemented batches, operated under OA of 2.0 g COD/L. The EEM spectrum was delineated into The fluorescence regions I (Ex/Em wavelengths: 200–250 nm/280–330 nm), II (Ex/Em wavelengths: 200–250/330–380 nm), III (Ex/Em wavelengths: 200–250/>380 nm), IV (Ex/Em wavelengths: >250/280–380 nm) and V (Ex/Em wavelengths: >250/>380 nm) were originated from tyrosine-like substances, biological oxygen demand content (mainly aromatic protein II), fulvic acid-like substances (FS), soluble microbial by-product (SMP)-like substances (tryptophan and protein-like groups) and humic-like substances (HS) [61]. It can be observed that the intensity of region V follows the following order, nFe_3_O_4_-supplemented > control > CNT-supplemented batch. This refers to the augmented generation of HS, in nFe_3_O_4_-supplemented batch, compared to the other two batches. This result is consistent with previous results that showed specific stimulation for HS, in the presence of nFe_3_O_4_ [62]. On the other, regions I, II, and IV in the EEM spectrum of CNT-supplemented batches had higher intensities than those in control batches. This indicates the enrichment of protein-like substances, in the presence of CNT [37,60]. Such HS and protein-like substances can work as shuttles for electrons, which could enhance the DIET pathway [53,61,62]. Previously, it was concluded that different CMs can enrich certain constituents of SMP [61]. Our finding agrees with this conclusion, further, referred to the enrichment of HS and protein-like substances, as a result of supplementing nFe_3_O_4_ and CNT, respectively.

### 3.3. Conductive Material-Based Microbial Community Change

**Figure 3:**
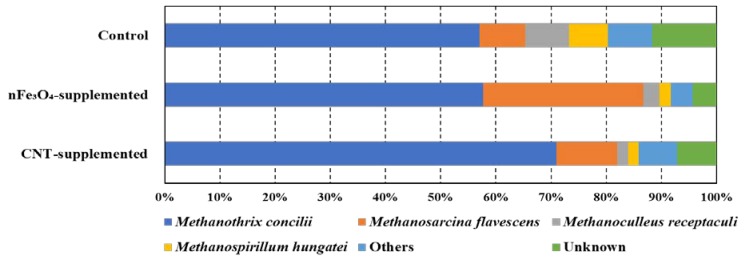
Microbial community structure at genus level for archaea acclimated to oleic acid in control, nFe_3_O_4_-supplemented, and CNT-supplemented batches.

To further confirm the positive effect of supplementing CM upon DIET pathway stimulation, microbial community analysis was carried out. In total, 104,137 high-quality reads of archaea were obtained. The average was 85,441 ± 5790 read count per sample after the clustering with CD-HIT-OUT. Strikingly, five genera accounted for 88% of the total sequences in the control, while percentages of over than 82% were presented by only two genera in nFe_3_O_4_- and CNT-supplemented batches (Figure 3).

Similarly, by stimulation of the DIET pathway, it was previously noted that the microbial diversity was decreased [44]. Figure 3 demonstrates that the abundance of *Methanothrix concilii* was 57%, 58%, 71% in the control, nFe_3_O_4_-supplemented, and CNT-supplemented batches, respectively. Further, the abundamce of *Methanosarcina flavescens* clearly increased in nFe_3_O_4_-supplemented batches (29%), compared to 8% and 11%, observed in control and CNT-supplemented batches, respectively. *Methanothrix* is an acetoclastic methanogen [46], while *Methanosarcina* can work as acetoclastic and hydrogentrophic [63]. Both of them are known as DIET-capable species [63,64]. High similarity of 99% was observed in *Methanothrix* and *Methanosarcina for M. concilii* and *M. flavescens*, respectively (Table 2).

**Figure 4:**
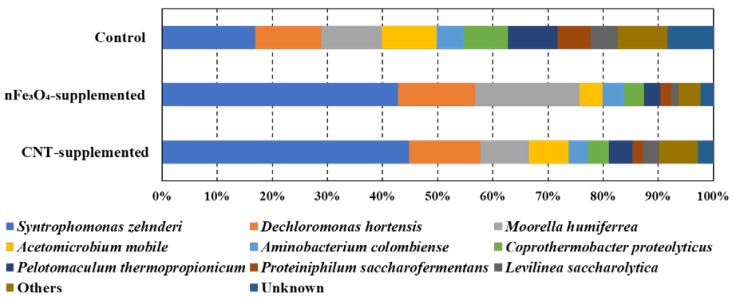
Microbial community structure at genus level for bacteria acclimated to oleic acid in control, nFe_3_O_4_-supplemented, and CNT-supplemented batches.

Figure 4 highlights the taxonomic abundance of the bacterial communities found in the three reactors. Totally, 22,917 ± 2338 high-quality reads of bacteria were obtained with an average of 39,989 ± 3424 read count per sample after the clustering with CD-HIT-OUT. Similarly, to archaea community change, 63% of identified bacteria, found in control, was represented by six genera, while 76% and 67% of the total bacteria abundance in nFe_3_O_4_-supplemented and CNT-supplemented batches, respectively, were occupied by only two genera.

As clearly depicted in Figure 4a, the abundance of *Syntrophomonas zehnderi* was increased from 17% (control) to 43% and 45% in nFe_3_O_4_-supplemented and CNT-supplemented batches, respectively. *S. zehnderi* is a well-known LCFA degrader [65], and the enrichment of *Syntrophomonas* after the supplementation of CM was reported before [2,65]. *Aminobacterium colombiense*, the amino acid degrader [66], and *Acetomicrobium mobile*, the acetate producer [67], were found in all batches with almost similar abundance. The highest abundances for *Dechloromonas hortensis* (14%) and *Moorella humiferrea* (19%) were found in nFe_3_O_4_-supplemented batches. *Dechloromonas hortensis* has the capability of using Fe (III) as electron acceptor [68], while *Moorella humiferrea* can grow through the electron shuttling between ferric ions and humic acid [69]. The similarity of *Syntrophomonas*, *Dechloromonas* and *Moorella* to *Syntrophomonas zehnderi*, *Dechloromonas hortensis* and *Moorella humiferrea* were 98% (Table 3). From this observation, it is clear that the supplementation of CNT and magnetite provided an effective environment for the growth of DIET-related syntrophic and methane producers.

## 4. Conclusions

In this study, various concentrations (0.1–4.0 g COD/L) of OA, as a model LCFA, were anaerobically digested in batch tests, while DIET reaction was stimulated by supplementing either nFe_3_O_4_ or CNT. The two supplements could overcome the toxicity of high OA concentration and increase CH_4_ production through two different mechanisms. Specifically, CNT supplementation could enhance sludge conductivity (121.1% higher than control) at an OA concentration of 2.0 g COD/L. At the same concentration, nFe_3_O_4_-supplemented batches showed significantly high dissolved iron concentration (3.2 times higher than control). The superiority of CNT over nFe_3_O_4_, in terms of enhancing CH_4_ productivity, might be due to the fact that raising sludge conductivity can be more vital, for digestion process efficacy, than iron concentration in broths. CNT supremacy can be also linked to the type of enriched soluble microbial by-products (protein-like substances), which are different from the humic-like substances that dominated nFe_3_O_4_-supplemented batches. Further, higher enrichment for *Syntrophomonas* sp., an electroactive bacterium, could be observed after CNT supplementation. This study concludes that DIET stimulation can be an ideal strategy for the efficient treatment of relatively high concentrations of potentially toxic substrates.

## Figures and Tables

**Table 1:** Batch anaerobic digestion performances at various OA concentrations and kinetic results obtained by using the modified Gompertz equation (r^2^ > 0.99).

Oleic Acid Concentration (g COD/L)	Additives	CH_4_ Potential (mL)	CH_4_ Production Rate (mL/d)	Lag Period (d)
0.10	Control	4.5	1.9	7.1
	nFe_3_O_4_	4.9	1.0	5.3
	CNT	5.1	1.6	6.0
0.25	Control	10.4	1.4	6.2
	nFe_3_O_4_	14.2	3.8	6.5
	CNT	13.4	2.3	6.4
0.50	Control	23.3	9.8	7.0
	nFe_3_O_4_	26.7	6.7	7.1
	CNT	28.1	8.0	7.1
1.00	Control	24.0	5.9	8.0
	nFe_3_O_4_	40.2	12.5	6.1
	CNT	49.2	9.5	6.6
2.00	Control	46.9	5.4	6.9
	nFe_3_O_4_	79.6	5.6	6.2
	CNT	96.2	28.6	7.1
4.00	Control	64.7	6.5	7.2
	nFe_3_O_4_	138.7	26.8	7.4
	CNT	171.9	23.6	7.1

**Table 2:** Archaeal communities in species level identification of the dominant sequences control, nFe_3_O_4_-supplemented and CNT-supplemented batches.

Microorganism	Accession Number	Similarity (%)	Control (%)	nFe₃O₄-Supplemented (%)	CNT-Supplemented (%)
*Methanothrix concilii*	NR_102903.1	99	57	58	71
*Methanosarcina flavescens*	NR_148758.1	99	8	29	11
*Methanoculleus receptaculi*	NR_043961.1	99	8	3	2
*Methanospirillum hungatei*	NR_074177.1	98	7	2	2

**Table 3:** Bacterial communities in species level identification of the dominant sequences of control, nFe_3_O_4_-supplemented, and CNT-supplemented batches.

Microorganism	Accession Number	Similarity (%)	Control (%)	nFe₃O₄-Supplemented (%)	CNT-Supplemented (%)
*Syntrophomonas zehnderi*	NR_044008.1	98	17	43	45
*Dechloromonas hortensis*	NR_042819.1	98	12	14	13
*Moorella humiferrea*	NR_108634.1	98	11	19	9
*Acetomicrobium mobile*	NR_102954.1	99	10	4	7
*Aminobacterium colombiense*	NR_074624.1	99	5	4	4
*Coprothermobacter proteolyticus*	NR_074653.1	100	8	4	4
*Pelotomaculum thermopropionicum*	NR_074685.1	98	9	3	4
*Proteiniphilum saccharofermentans*	NR_148807.1	99	6	2	2
*Levilinea saccharolytica*	NR_040972.1	99	5	1	3
